# Effects of Visual Perturbation on Single-Leg Drop Jump Biomechanics in Patients Post-Anterior Cruciate Ligament Reconstruction

**DOI:** 10.3390/jcm15010118

**Published:** 2025-12-24

**Authors:** Xavier Laurent, Damien Dodelin, Nicolas Graveleau, Nicolas Bouguennec

**Affiliations:** 1 Restart, Medical Stadium, 33700 Mérignac, France; 2 Biomechanical Laboratory, Medical Stadium, 33700 Mérignac, France; 3 Clinique du Sport, 33700 Mérignac, France

**Keywords:** anterior cruciate ligament reconstruction, biomechanics, neurodynamics, visual perturbation, lower extremity

## Abstract

**Background**: Patients after anterior cruciate ligament reconstruction (ACLR) often exhibit persistent biomechanical deficits, particularly during high-demand tasks like the single-leg drop jump (SLDJ). At approximately six months post-ACLR, patients frequently rely on visual input to compensate for persistent sensorimotor deficits during dynamic tasks, which may lead to altered movement patterns. While visual perturbations have been studied in bilateral jump tasks, their impact on SLDJ biomechanics in ACLR patients remains unexplored. **Methods**: Patients who were still engaged in rehabilitation and not yet cleared for unrestricted return to sport performed SLDJ under three visual conditions: normal vision, low visual perturbation, and high visual perturbation using stroboscopic glasses. Kinematic and kinetic variables were measured using a 3-dimensional motion analysis system and force platform. Comparisons were made between the ACLR and non-operated limbs, as well as across visual conditions. **Results**: 24 patients (17 males, 7 females; mean age 25.6 ± 6.3 years, mean height 174 ± 9.0 cm, mean weight 74.7 ± 17.2 kg) were included in the analysis. Knee adduction excursion during landing was significantly affected by visual perturbation (F(2, 46) = 6.55, *p* = 0.004, η^2^ = 0.019). Post hoc analysis showed that high visual perturbation significantly decreased knee adduction excursion compared to normal vision on the ACLR limb (mean difference 1.499°, SE = 0.388, pBonf = 0.003, Cohen’s d = 0.542). A significant difference was also found between low and high visual perturbation on the ACLR limb (mean difference 1.543°, SE = 0.388, pBonf = 0.002, Cohen’s d = 0.558). No significant changes were observed in the non-operated limb across visual conditions. **Conclusions**: High visual perturbation significantly altered knee adduction excursion on the ACLR limb, resulting in a shift toward greater knee abduction during landing. No changes were observed in the non-operated limb. These findings support the use of visual perturbation in functional assessment protocols after ACLR to better identify persistent biomechanical deficits that may contribute to reinjury risk.

## 1. Introduction

Anterior cruciate ligament (ACL) tears are a common orthopedic problem, particularly among athletes engaged in high-intensity sports that require rapid changes in direction, abrupt stops, and jumps [1]. Surgical reconstruction of the ACL has become the gold standard treatment to restore knee stability and to allow a high rate of return to sports [2]. However, despite advances in surgical techniques and post-operative rehabilitation protocols, a significant percentage of patients continue to exhibit persistent functional deficits [3,4]. Around six months after ACLR, many patients have recovered functional abilities such as running and single-leg hopping, yet continue to demonstrate residual neuromuscular and sensorimotor deficits. This stage typically corresponds to the first structured return to sport evaluation, but not to unrestricted sports participation. Therefore, this timepoint represents a clinically relevant moment to assess movement patterns and identify persistent biomechanical or sensorimotor deficits that may not be detectable under standard testing conditions. 

Biomechanical assessments of patients after ACL reconstruction (ACLR) often reveal asymmetries in lower limb kinematics and kinetics, which may contribute to an increased risk of reinjury, especially during sports movements such as drop jumps [5,6]. The single-leg drop jump (SLDJ) is a particularly demanding biomechanical task, requiring complex coordination between visual perception, postural stability, and the ability of the neuromuscular system to absorb and redistribute impact forces. Furthermore, single-leg tests are useful to assess each limb capacity, whereas double-leg tests offer the opportunity to analyze interlimb compensatory strategies [7]. Patients with ACLR often demonstrate altered landing mechanics characterized by reduced knee flexion alongside increased hip flexion, ankle plantarflexion, anterior pelvic tilt and trunk flexion. These patterns are considered compensatory strategies aimed at reducing knee joint loading in response to persistent knee extension weakness and impaired sensorimotor control, rather than injury-causing mechanics. However, such compensations may contribute to suboptimal frontal plane control during dynamic tasks [8]. Thus, several studies have highlighted the importance of adding vertical tests during the return to sport evaluation after ACLR [7,8,9]. 

Visual perturbations, such as reduced visual field or alterations in depth perception, are factors that can exacerbate biomechanical deficits in patients with ACLR. Several studies have shown that vision plays a crucial role in postural control and dynamic stability, particularly under dual-task conditions where the visual system compensates for proprioceptive deficits in ACLR conditions [10,11]. Thus, incorporating visual perturbation into post-ACLR assessment protocols may help clinicians better identify persistent biomechanical and sensorimotor deficits that are not apparent under normal visual conditions [12,13,14]. Despite the recognized importance of vision in motor control, only a few studies have specifically examined the impact of visual perturbations on the biomechanics of ACLR patients during double-leg drop landing or cutting mechanics. They reported decreased knee flexion excursion and reduced peak knee extension moments under visual perturbation conditions [15,16]. It is therefore crucial to understand how these perturbations may affect lower limb kinematic and kinetic parameters and whether they alter the landing strategies adopted by these patients. The observed deficits could have direct implications for the development of targeted rehabilitation programs aimed at enhancing dynamic stability and preventing injuries. To our knowledge, no previous study has evaluated the impact of visuomotor perturbation on SLDJ biomechanics in ACLR patients. 

The aim of this study was to investigate the effects of graded visual perturbation on single-leg drop jump biomechanics in patients approximately six months after anterior cruciate ligament reconstruction. Specifically, we examined whether increasing levels of visual disturbance would differentially affect knee kinematics and kinetics between the reconstructed and non-operated limbs. We hypothesized that higher levels of visual perturbation could reveal limb-specific alterations in frontal-plane knee control on the ACLR side that are not apparent under normal visual conditions. By addressing this question at a clinically relevant return to sport timepoint, this study seeks to inform future research exploring visuomotor-based assessment strategies and their potential role in guiding rehabilitation progression after ACL reconstruction.

## 2. Methods

### 2.1. Study Design

This study used an observational, cross-sectional, repeated-measures design. Each participant completed a standardized single-leg drop jump protocol under three visual conditions. A within-subject comparison framework was used, enabling each participant to be assessed on both limbs (ACLR and healthy) across all visual conditions.

### 2.2. Study Participants

Participants were included if they had undergone ACLR. The inclusion criteria included: medical clearance from the surgeon following a clinical evaluation; resumption of running during rehabilitation; ability to perform vertical and horizontal single-leg hops without pain; passive and active range of motion >90% compared to the contralateral side; no inflammatory response or joint edema in the past 15 days; and having a goal of returning to sport. Patients undergoing a second ACLR, those with multi-ligament injuries, and patients >223 days (7.5 months) post-surgery were excluded. Sports participation was assessed using the Tegner score, which is a scale ranging from 0 to 10 used to assess an individual’s level of physical and sports activity [17].

A total of 26 participants (18 males and 8 females) were included and tested. After demographic analysis, two patients were subsequently excluded as they did not meet the inclusion criteria and were too long post-surgery (286 and 311 days). Thus, 24 individuals (17 males, 7 females) were finally analyzed. No a priori sample size estimation was performed because this study was designed as an exploratory within-subject biomechanical analysis, consistent with prior ACLR visuomotor research using similar sample sizes (n = 10–20) [15,16]. The sample size of 24 participants was determined by the number of eligible patients during the recruitment period and is in line with published motion-analysis studies conducted at comparable postoperative timepoints. 

The mean (SD) age of the 24 participants was 25.6 ± 6.3 years (range: 16–39). Mean (SD) height was 174 ± 9.0 cm and mean (SD) weight was 74.7 ± 17.2 kg. All participants underwent ipsilateral semitendinosus autograft reconstruction surgery. 

Mean (SD) post-operative period was 187.6 ± 15.5 days, corresponding to a mean of 6.2 months (Table 1). At the time of testing, none of the participants had returned to unrestricted sports participation. However, all were engaged in a structured return-to-sport rehabilitation program and had resumed straight-line running and basic plyometric activities, as required by the inclusion criteria. Before injury, participants were recreational to competitive athletes (Tegner score 7.38 ± 1.84), mainly involved in soccer, handball, rugby, or similar cutting/pivoting sports. Their sports activity level during testing corresponded to early return-to-sport rehabilitation rather than full sport participation. Regarding the operated side, 15 participants (62.5%) had surgery on the right side and nine (37.5%) on the left side. Mean (SD) pre-injury Tegner score was 7.38 ± 1.84. 

### 2.3. Assessment Procedure

The study participants underwent three-dimensional (3D) motion analysis tests conducted at the same location by the same examiner. Participants wore sports shorts and standard shoes. Prior to testing, all participants completed a 5 min warm-up session consisting of running, deep squats, lunges, and hops, supervised by a physiotherapist. 

For the purpose of this study, only the single-leg drop jump task was analyzed. Although participants underwent a broader clinical assessment as part of their routine rehabilitation follow-up, only the SLDJ with and without visual perturbation was included in the present research protocol.

This study focused on evaluating unilateral drop jumps. The SLDJ was performed from a 20 cm step, as reported previously [18]. A physiotherapist provided verbal instructions during the tasks. Subjects were instructed to drop from the step and, upon landing, immediately jump as high as possible while minimizing ground contact time on the force platform [8]. During the task, the participants kept their hands on their hips for consistency. All SLDJ trials were performed unilaterally. Each participant completed the task on both limbs separately (ACLR and healthy), and three successful trials were retained for the analysis. The order of limb testing (ACLR and healthy) was randomized across participants. The SLDJ was performed under three conditions: full vision, low-level stroboscopic visual perturbation, and high-level stroboscopic visual perturbation. The order of visual conditions was not randomized, as the stroboscopic perturbation required a fixed progression from full vision to low and then high visual disturbance to ensure proper accommodation. Three trials were completed for each condition, and the average was calculated for each participant. Before testing with stroboscopic perturbation, participants completed an accommodation protocol consisting of a 5 min ball-throwing exercise where the visual perturbation rate increased after every set of five successful catches, allowing accommodation to the stroboscopic visual perturbation and reducing novelty effects, as reported previously [16,19]. Participants completed at least two practice trials before each condition. Stroboscopic glasses (Senaptec, Beaverton, OR, USA) imposed the visual perturbation conditions. These glasses, which are similar to sunglasses, are equipped with a strap for secure fitting and lenses with liquid crystal displays powered by batteries. They do not block vision continuously, but emit black flashes to block vision for a few milliseconds (ms) at a time. The duration and frequency of these “lost” and “intact” vision periods can be customized across eight levels, with constant transparent periods of 100 ms and opaque periods ranging from 67 to 900 ms. Two levels of perturbation were used: low visual perturbation, referred to as Glasses 1 (opaque 100 ms, transparent 100 ms), and high visual perturbation, referred to as Glasses 2 (opaque 344 ms, transparent 100 ms). Participants first completed all trials in full vision, followed by stroboscopic visual perturbation trials until three successful trials were captured for each lower limb.

Kinematic and kinetic variables were captured during the SLDJ tasks. Kinematic data were tracked using a 3D motion analysis system (Miqus M3; Qualisys, Gothenburg, Sweden) comprising 10 optoelectronic cameras sampled at 240 Hz. Each subject was fitted with 47 retroreflective markers (Super-Spherical markers; Qualisys, Gothenburg, Sweden) positioned according to the dynamic “Qualisys Sports Marker Set” model [20]. These markers were secured with transparent double-sided tape to prevent detachment. To ensure optimal adherence, marker areas were sprayed with a sticky adhesive beforehand. Once all markers were correctly positioned, the athlete stood on a force platform. Kinetic data were captured simultaneously using an integrated force platform measuring 60 cm × 90 cm (AMTI; Advanced Mechanical Technology, Inc., BMS600900 1000 Hz, Watertown, NY, USA), sampled at 1000 Hz.

Biomechanical data analysis was performed using visual 3D software (v6.03.3 Professional; C-Motion, Inc., Germantown, MD, USA), allowing extraction of kinematic, kinetic, and inverse dynamic variables. The variables of interest (jump height, ground contact time, flight time, peak vertical ground reaction force, peak knee extensor moment, peak knee power absorption, peak knee power generation, peak knee flexion, landing knee flexion excursion, landing peak knee abduction moment, and landing knee adduction excursion, were extracted using a custom Python script (Python Software Foundation, version 3.9.7; Wilmington, DE, USA) and an Excel spreadsheet (Microsoft 365 Apps for Enterprise, version 2311; Microsoft Corporation, Redmond, WA, USA). 

### 2.4. Statistical Analysis 

All analyses were performed using JASP Team (2024), version 0.18.3. Most of the variables analyzed in this study followed a normal distribution, as determined by the Shapiro–Wilk test; however, some variables showed deviations from normality including: landing knee flexion excursion under full vision conditions (*p* = 0.033), landing peak knee abduction moment under glasses 2 conditions (*p* = 0.027), peak vertical ground reaction force under full vision conditions (*p* < 0.001), ground contact time across visual conditions (*p* < 0.001 full vision, *p* = 0.026 glasses 1, *p* = 0.012 glasses 2), and peak knee power absorption under full vision (*p* < 0.001). 

Repeated-measures ANOVA was retained to compare kinematic and kinetic variables across limbs because this method is considered robust to moderate violations of normality in within-subject designs, particularly when sphericity is addressed [21,22,23,24]. When the sphericity assumption was violated, the Greenhouse-Geisser correction was applied. Significant effects were explored using Bonferroni-adjusted post hoc comparisons to determine which specific conditions exhibited significant differences. A significance threshold of *p* < 0.05 was set. 

Effect sizes were reported to quantify the magnitude of observed differences. Eta-squared (η^2^) was used to evaluate the proportion of total variance attributable to a factor in the repeated-measures ANOVA. η^2^ values are interpreted as follows: small (η^2^ = 0.01), moderate (η^2^ = 0.06), and large (η^2^ ≥ 0.14) [25]. Additionally, Cohen’s d was used to compare differences in post hoc analyses between visual conditions and limbs. Cohen’s d values are interpreted as follows: small (d = 0.2), moderate (d = 0.5), and large (d ≥ 0.8) [26]. Variables that did not meet normality assumptions are interpreted with caution, as noted in the Discussion section.

## 3. Results

A Repeated-measures ANOVA with two within-subject factors, Visual Condition (full, low, high) and Limb (ACLR vs. Healthy), was performed for each biomechanical outcome. Among all measured variables, only landing knee adduction excursion demonstrated significant effects, as detailed below.

Analysis of the effect of the visual condition found significant results only for knee adduction excursion during landing, with a small to moderate effect size (F(2, 46) = 6.55; *p* = 0.004 **; η^2^ = 0.019). Descriptive data for the different variables are shown in Table 2.

Analysis of the interaction effect between visual condition and limb showed a significant difference, again with a small to moderate effect size, for knee adduction excursion during landing (F(2, 46) = 3.88; *p* = 0.031; η^2^ = 0.011) (Table 2).

Regarding the post hoc analyses for the visual condition, the difference between normal vision and Glasses 1 was not statistically significant (pBonf = 1.00). However, the mean difference between normal vision and Glasses 2 was 0.861° (SE = 0.274, t = 3.136, Cohen’s d = 0.311, pBonf = 0.007 **), and the mean difference between Glasses 1 and Glasses 2 was 0.860° (SE = 0.274, t = 3.133, Cohen’s d = 0.275, pBonf = 0.007 **) (Table 3). These results indicate that knee adduction excursion during landing is significantly altered during high visual disturbance (Glasses 2) compared to normal vision and low visual disturbance (Glasses 1), with a moderate effect size. 

For the post hoc analysis of the visual condition * limb interaction, specific post hoc comparisons showed that between the ACLR limb with normal vision and the ACLR limb with Glasses 1 visual perturbation, the mean difference was −0.044 (SE = 0.388, t = −0.114, pBonf = 1.000), changing from −0.082 ± 2.617° to −0.038 ± 3.019°, indicating a non-significant reduction in negative knee adduction excursion during landing (i.e., a reduction in knee abduction excursion) under low visual disturbance compared to normal vision. Between the ACLR limb with normal vision and the ACLR limb with Glasses 2, the mean difference was 1.499° (SE = 0.388, t = 3.861, pBonf = 0.003 **), changing from a mean (SD) of −0.082 ± 2.617° to −1.580 ± 2.486°, indicating a significant increase in knee abduction excursion during landing under high visual disturbance compared to normal vision. The effect size for this comparison was moderate (d = 0.542). Between the ACLR limb with Glasses 1 and the ACLR limb with Glasses 2, a significant mean difference of 1.543° (SE = 0.388, t = 3.975, pBonf = 0.002 **) was detected, changing from a mean of −0.038 ± 3.019° to −1.580 ± 2.486°, indicating a significant increase in knee abduction excursion during landing under high visual disturbance compared to low visual disturbance. The effect size for this comparison was moderate (d = 0.558). Between the healthy limb with normal vision and the healthy limb with Glasses 1, the observed difference was 0.046° (SE = 0.388, t = 0.118, pBonf = 1.000), which was not significant. Between the healthy limb with normal vision and the healthy limb with Glasses 2, the observed difference was also not significant at 0.223° (SE = 0.388, t = 0.574, pBonf = 1.000). Finally, between the healthy limb with Glasses 1 and the healthy limb with Glasses 2, the mean difference was 0.177° (SE = 0.388, t = 0.456, pBonf = 1.000), indicating no significant difference between visual disturbance levels on the healthy limb

No significant differences were observed between visual conditions on the healthy limb, as detailed in Table 4. Table 4 reports all pairwise contrasts produced by the limb x visual condition interaction model. Only within-limb comparisons across visual conditions directly address the study objectives; between-limb contrasts are included for completeness but are not interpreted as effects of visual perturbation.

**Figure 1:**
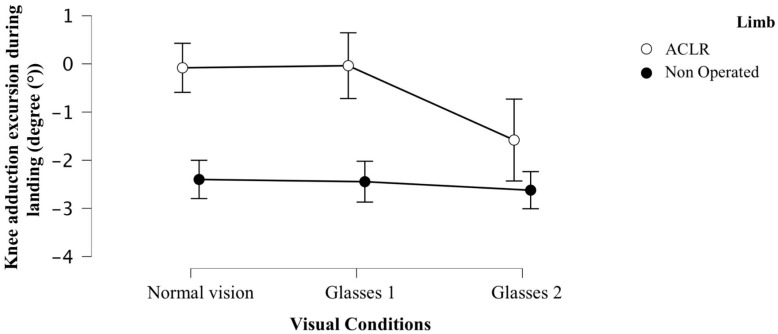
Knee adduction excursion during landing (in degrees) for the different visual conditions and limbs.

These results highlight that only the operated limb (ACLR) is influenced by the visual condition, while the healthy limb remains relatively stable across the different visual conditions. The high level of visual perturbation (Glasses 2) increases knee abduction excursion during landing in the operated limb (Figure 1).

## 4. Discussion

The use of visual perturbation in this study revealed significant alterations of SLDJ biomechanics in patients 6 months post-ACLR. These results suggest that visual disturbance, particularly under high disturbance conditions, significantly alters knee adduction excursion during landing in the operated limb. Specifically, the high visual disturbance condition resulted in a significant mean increase of 1.499° in knee abduction excursion compared to the normal vision test condition, with a moderate effect size (*p* = 0.003, d = 0.542). This difference exceeds the minimum detectable threshold (0.98°) previously reported for this variable in a double-leg task [16]. Because the present study did not include a test–retest design, reliability indices such as the intraclass correlation coefficient could not be calculated for our dataset and previously published minimum detectable threshold values were used for contextual interpretation. In contrast, knee adduction excursion on the healthy side was not significantly affected by visual disturbance.

Grooms et al. previously reported that visual disturbance via stroboscopic glasses increased sagittal and frontal knee excursion during a bilateral drop landing task in individuals with ACLR [16]. However, they did not observe any inter-limb differences, nor differences between ACLR and matched controls for frontal plane knee excursion. Their visual-perturbation effect on frontal plane knee excursion corresponded to a mean change of 1.98°, with a small and non-significant between-group effect size (r = 0.18, *p* = 0.49). In contrast, our unilateral SLDJ at approximately 6 months post-ACLR revealed a moderate limb-specific effect of high visual perturbation on landing knee adduction excursion (Cohen’s d = 0.542) in the operated limb only. Differences in task demands (unilateral vs. bilateral) and post-operative timing 6 months vs. 36.2 months in Grooms et al.) likely explain why inter-limb asymmetries were detected in our study but not in theirs, as later postoperative stages may allow greater recovery of symmetry. 

Neuroimaging studies have reported various consequences associated with ACLR that may help explain the results observed in this study. First, increased neuronal activity in different cortical areas measured by functional magnetic resonance imaging (fMRI) has been reported in ACLR patients compared to healthy individuals during multi-joint motor control tasks involving the hip and knee [25]. Increased activity was first observed in the superior parietal lobule, which plays a crucial role in spatial orientation and perception, suggesting increased spatial processing needs for motor control in ACLR patients [26], and then in the parietal lobe, which could indicate reduced efficiency in processing spatial information [27], and additionally in several occipital regions, particularly the lingual gyrus, a multimodal area that uses feedback projections to the parietal cortex for multisensory integration of visual and somatosensory information [28]. Finally, in regions near the parieto-temporo-occipital junction, indicating a disruption of visual perception of movement and limbs in ACLR patients [29]. Moreover, functional connectivity analyses via fMRI have also reported extensive connectivity between sensorimotor regions in ACLR patients, suggesting that increased spatial information processing may be involved in motor coordination in these patients [30].

A recent case–control study investigated the overlapping and unique brain and cerebellar activities for motor control of the injured and uninjured knee during a single-joint knee flexion-extension task between ACLR and control groups [30]. The authors reported unique activations in the precuneus, an area involved in sensory integration, for the injured limb compared to the contralateral limb or control group. The precuneus has already been identified as a multisensory region responsible for directing spatial attention during movement execution and visual cognition [31,32]. While other regions demonstrated altered bilateral cerebellar activities compared to controls, the findings of this study tend to indicate a compensatory neural strategy to maintain motor control, particularly for the injured side.

Previous electroencephalography (EEG) research in ACLR patients reported higher frontal theta frequency activation during a force control task compared to control subjects, suggesting increased cognitive control of movement or loss of cerebellar automaticity [33]. Individuals after ACLR may therefore rely more on cortical control than uninjured individuals, who utilize a strategy more regulated by the cerebellum to perform the same knee movements [30,33]. 

These, and previously reported, data expose a form of sensory reweighting in ACLR patients [25,34,35]. Typically, sensory systems (somatosensory, vestibular, visual) are dynamically organized. The level of dependence on each system is adjusted according to different circumstances and environmental demands by weighting more reliable sensory information to maintain motor control [36]. In the case of an ACL injury, damaged mechanoreceptors impair the sensitivity and acuity of proprioceptive signals from the knee, inducing strategies of neural reorganization to maintain sufficient sensory feedback for coordinating lower limb movements. More specifically, these neural strategies may reduce the weighting of proprioceptive information and increase that of other sensory modalities, such as visual information. Thus, the increase in cortical activity among visual and multimodal regions may be a central compensatory response to ensure accurate spatial orientation and limb coordination: a form of strategy and/or visual-motor dependence in motor control [25,30].

The observed alterations in the operated knee biomechanics in the frontal plane under high visual disturbance conditions in our study indicate that landing biomechanics may be influenced by the amount of visual feedback. Specifically, when visual feedback is not greatly disturbed, patients manage to maintain similar neuromuscular control to the normal vision condition. However, when visual feedback is highly disturbed, they lose motor control of the knee in the frontal plane, leading to an increase in knee abduction, particularly on the operated side. This finding has practical relevance as physical activity and sports participation impose high demands on the visual-motor system to maintain interaction with the environment as well as neuromuscular control [37]. In a sports context, the capabilities of the visual system may thus be more dedicated to strategic, tactical, or technical sports objectives rather than ensuring joint motor control, particularly in multidirectional team sport athletes. These athletes are moreover prone to numerous ACL injuries [1]. Given that non-contact ACL injury (or reinjury) can be considered as a neurocognitive error [38,39], assessing and rehabilitating central and visuomotor ACLR consequences using visual disturbances would be relevant. Recent promising findings align with the development of these specific rehabilitation techniques and plans [14]. 

These findings also raise rehabilitation considerations, although the present study was not designed to test an intervention. The presence of visuomotor alterations at six months suggests that such deficits may persist despite standard rehabilitation. Whether they result from early compensations, limited proprioceptive stimulation, or other neurophysiological adaptations cannot be determined here. However, identifying these deficits at a clinically relevant timepoint indicates that earlier sensory-challenging progressions could be explored in future work. 

In practical terms, our results support visual perturbation primarily as an assessment tool rather than a therapeutic approach. High-level perturbation (344 ms opaque) was the only condition that revealed meaningful alterations, suggesting its utility for detecting deficits that are not apparent under normal vision. Whether integrating visuomotor perturbation into training tasks (e.g., SLDJ progressions) improves biomechanical control remains unknown and should be addressed in future interventional studies. At this stage, visual perturbation helps refine diagnostics but does not allow us to prescribe a specific rehabilitation protocol. 

It is, however, important to specify that our patients were evaluated at an average of 6 months post-surgery, while most studies evaluating the central consequences post-ACLR assess patients at 24 or 36 months post-surgery [25,31,40,41]. This earlier postoperative timepoint was intentionally selected because six months corresponds to a typical structured return to sport evaluation in clinical practice. At this stage, patients have typically regained basic functional capacities (e.g., running, hopping, jumping) but still present meaningful sensorimotor and neuromuscular deficits that influence landing mechanics and decision-making regarding sport resumption. Studying visuomotor disturbances at this clinically relevant milestone, therefore, allows identification of deficits that may directly impact rehabilitation progression and reinjury risk, complementing prior work that explored longer-term neural adaptations at 24–36 months post-ACLR. Notably, our findings suggest that visuomotor consequences are already present at 6 months post-surgery, highlighting the need for longitudinal assessments to better understand their evolution across recovery. Moreover, the increase in knee abduction excursion on the operated side under visual disturbance level 2 in our study did not exceed the knee abduction excursion of the uninjured side, which remained relatively stable, but higher, depending on the visual condition. It is possible that the tested patients, still in rehabilitation, were frequently trained to control the abduction of the operated knee by their physiotherapist since correcting knee abduction movement is part of rehabilitative goals and return-to-sport criteria [42,43,44]. This may have potentially influenced our results. The inter-limb repeated-measures design used in this study reduces inter-individual variability but does not fully exclude the influence of limb dominance. Future studies, including matched healthy controls, would help isolate visuomotor deficits that are specifically attributable to ACLR. Some secondary variables did not meet normality assumptions, and although repeated-measures ANOVA is considered robust in this context, these specific outcomes should be interpreted with caution. Future studies may complement these analyses with non-parametric or permutation-based approaches to confirm these findings. Finally, we mainly focused our evaluations on knee kinetics and kinematics. It is commonly reported that ACLR patients exhibit trunk, pelvic, hip, or ankle compensatory mechanics that may influence knee excursion [7,18,45,46]. 

## 5. Conclusions

Visual disturbance through stroboscopic glasses significantly alters knee biomechanics in patients post-ACLR, particularly under high disturbance conditions on the involved leg. These results indicate that visual perturbation may serve as a valuable assessment tool for uncovering visuomotor deficits that are not apparent under normal visual conditions. More specifically, the use of high levels of visual disturbance appears necessary to elicit meaningful changes in SLDJ mechanics at this stage of recovery.

## Figures and Tables

**Table 1:** Descriptive data for the study population at inclusion.

	Mean	Standard Deviation	Minimum	Maximum
Age (years)	25.6	6.3	16	39
Height (cm)	174	9.0	159	191
Weight (kg)	74.7	17.2	52	116
Time post-surgery (days)	187.6	15.5	162	223
Tegner score pre-injury	7.38	1.84	4	10

**Table 2:** Results of repeated measures ANOVA for the different variables.

SLDJ Variables	Visual Condition			Interaction Condition Visual *-Limb		
	*p* Value	Fisher’s F	Effect Size (η^2^)	*p* Value	Fisher’s F	Effect Size (η^2^)
Jump height	0.442	F (2, 46) = 0.24	0.004	0.614	F (1, 46) = 0.43	0.002
Ground contact time	0.095	F (2, 46) = 2.41	0.006	0.907	F (1, 46) = 0.08	0.0002
Flight time	0.328	F (2, 46) = 0.98	0.014	0.328	F (1, 46) = 0.98	0.014
Peak vertical ground reaction force	0.173	F (2, 46) = 1.90	0.018	0.220	F(1, 46) = 1.56	0.014
Peak knee extensor moment	0.881	F (2, 46) = 0.13	0.0002	0.885	F (1, 46) = 0.11	0.0002
Peak knee power absorption	0.155	F (2, 46) = 1.91	0.005	0.539	F (1, 46) = 0.60	0.002
Peak knee power generation	0.932	F (2, 46) = 0.07	0.00008	0.080	F (1, 46) = 2.61	0.003
Peak knee flexion	0.246	F (2, 46) = 1.43	0.003	0.603	F (1, 46) = 0.41	0.0008
Landing knee flexion excursion	0.160	F (2, 46) = 1.94	0.005	0.899	F (1, 46) = 0.07	0.0002
Landing peak knee abduction moment	0.370	F (2, 46) = 0.98	0.00098	0.436	F (1, 46) = 0.80	0.0008
Landing knee adduction excursion	0.004 **	F (2, 46) = 6.55	0.019	0.031 *	F (1, 46) = 3.87	0.011

* *p* < 0.05, ** *p* < 0.01.

**Table 3:** Post hoc analysis of landing knee adduction excursion variation under the different visual conditions.

		Mean Difference	Standard Error	t	Cohen’s d	pBonf
Normal vision	Glasses 1	8.333 × 10^−4^	0.274	0.003	3.013 × 10^−4^	1.000
	Glasses 2	0.861	0.274	3.136	0.311	0.007 **
Glasses 1	Glasses 2	0.860	0.274	3.133	0.311	0.007 **

** *p* < 0.01. The *p*-value was adjusted for comparing a family of three. The mean results are shown over the levels of limb.

**Table 4:** Post hoc analysis of landing knee adduction excursion—Interaction limb * visual condition.

		Mean Difference	Standard Error	t	Cohen’s d	pBonf
ACLR, normal vision	Healthy, normal vision	2.317	0.798	2.902	0.838	0.076
	ACLR, glasses 1	−0.044	0.388	−0.114	−0.016	1.000
	Healthy, glasses 1	2.363	0.798	2.960	0.854	0.065
	ACLR, glasses 2	1.499	0.388	3.861	0.542	0.003 **
	Healthy, glasses 2	2.540	0.798	3.182	0.918	0.034 *
Healthy, normal vision	ACLR, glasses 1	−2.362	0.798	−2.958	−0.854	0.065
	Healthy, glasses 1	0.046	0.388	0.118	0.017	1.000
	ACLR, glasses 2	−0.819	0.798	−1.025	−0.296	1.000
	Healthy, glasses 2	0.223	0.388	0.574	0.081	1.000
ACLR, glasses 1	Healthy, glasses 1	2.408	0.798	3.015	0.870	0.055
	ACLR, glasses 2	1.543	0.388	3.975	0.558	0.002 **
	Healthy, glasses 2	2.585	0.798	3.237	0.934	0.029 *
Healthy, glasses 1	ACLR, glasses 2	−0.865	0.798	−1.083	−0.313	1.000
	Healthy, glasses 2	0.177	0.388	0.456	0.064	1.000
ACLR, glasses 2	Healthy, glasses 2	1.042	0.798	1.305	0.377	1.000

* *p* < 0.05, ** *p* < 0.01. *p*-value adjusted for comparing a family of 15.

## Data Availability

The datasets generated and analyzed during the current study are not publicly available due to privacy and CNIL (French data protection authority) restrictions but are available from the corresponding author upon reasonable request.

## Abbreviations

ACL	Anterior Cruciate Ligament
ACLR	Anterior Cruciate Ligament Reconstruction
ANOVA	Analysis of Variance
EEG	Electroencephalography
fMRI	Functional Magnetic Resonance Imaging
GC	Ground Contact
GRF	Ground Reaction Force
IC	Initial Contact
MDC	Minimal Detectable Change
RTS	Return to Sport
SD	Standard Deviation
SE	Standard Error
SLDJ	Single-Leg Drop Jump
VGRF	Vertical Ground Reaction Force
